# Chromatin damage generated by DNA intercalators leads to degradation of RNA Polymerase II

**DOI:** 10.1093/nar/gkae069

**Published:** 2024-02-10

**Authors:** Jaime A Espinoza, Dimitris C Kanellis, Sheetanshu Saproo, Karla Leal, Johana Fernandez Martinez, Jiri Bartek, Mikael S Lindström

**Affiliations:** Science for Life Laboratory, Division of Genome Biology, Department of Medical Biochemistry and Biophysics, Karolinska Institutet, S-171 21 Stockholm, Sweden; Science for Life Laboratory, Division of Genome Biology, Department of Medical Biochemistry and Biophysics, Karolinska Institutet, S-171 21 Stockholm, Sweden; Science for Life Laboratory, Division of Genome Biology, Department of Medical Biochemistry and Biophysics, Karolinska Institutet, S-171 21 Stockholm, Sweden; Science for Life Laboratory, Division of Genome Biology, Department of Medical Biochemistry and Biophysics, Karolinska Institutet, S-171 21 Stockholm, Sweden; Science for Life Laboratory, Division of Genome Biology, Department of Medical Biochemistry and Biophysics, Karolinska Institutet, S-171 21 Stockholm, Sweden; Science for Life Laboratory, Division of Genome Biology, Department of Medical Biochemistry and Biophysics, Karolinska Institutet, S-171 21 Stockholm, Sweden; Danish Cancer Society Research Center, DK-2100 Copenhagen, Denmark; Science for Life Laboratory, Division of Genome Biology, Department of Medical Biochemistry and Biophysics, Karolinska Institutet, S-171 21 Stockholm, Sweden

## Abstract

In cancer therapy, DNA intercalators are mainly known for their capacity to kill cells by inducing DNA damage. Recently, several DNA intercalators have attracted much interest given their ability to inhibit RNA Polymerase I transcription (BMH-21), evict histones (Aclarubicin) or induce chromatin trapping of FACT (Curaxin CBL0137). Interestingly, these DNA intercalators lack the capacity to induce DNA damage while still retaining cytotoxic effects and stabilize p53. Herein, we report that these DNA intercalators impact chromatin biology by interfering with the chromatin stability of RNA polymerases I, II and III. These three compounds have the capacity to induce degradation of RNA polymerase II and they simultaneously enable the trapping of Topoisomerases TOP2A and TOP2B on the chromatin. In addition, BMH-21 also acts as a catalytic inhibitor of Topoisomerase II, resembling Aclarubicin. Moreover, BMH-21 induces chromatin trapping of the histone chaperone FACT and propels accumulation of Z-DNA and histone eviction, similarly to Aclarubicin and CBL0137. These DNA intercalators have a cumulative impact on general transcription machinery by inducing accumulation of topological defects and impacting nuclear chromatin. Therefore, their cytotoxic capabilities may be the result of compounding deleterious effects on chromatin homeostasis.

## Graphical Abstract

**Graphical Abstract:**
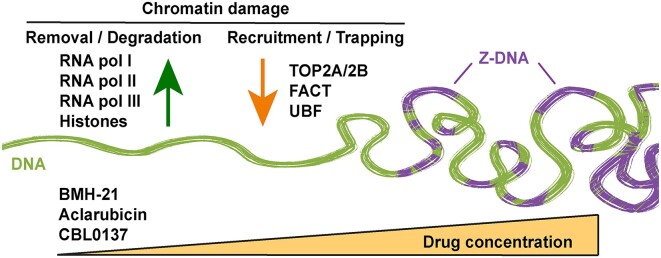

## Introduction

Targeting DNA through conventional chemotherapy is still a widely used strategy for cancer therapy (1), and there are continuous efforts to find more specific ways to harness the DNA damage response to eradicate cancer (2). Although the detrimental impact of DNA damage on cell viability is established, in part by the early success of alkylating agents and topoisomerase inhibitors (2), less attention has been drawn to how DNA-targeting drugs affect chromatin, seen as a complex network of nucleoproteins with an array of processes taking place on it simultaneously (3). In principle, a DNA intercalator is a type of drug that is capable of interfering with DNA topology and/or inducing DNA damage. There are several hundred known intercalators that can be classified into a few of compound classes, e.g. ellipticines and acridines (4).

Although several clinically approved and experimental DNA intercalators have been reported to show seemingly specific properties, less is known about the common ways by which they impact chromatin. Chromatin can adopt different levels of compaction and higher-order structures that influence gene accessibility and regulation. Changes in chromatin structure, such as the loss of higher-order organization or the formation of aberrant chromatin structures, can impact gene expression and genomic stability. In this setting, the broader concept of ‘chromatin damage’ has been used and refers to the structural and functional alterations that occur not only to DNA but also to protein and RNA molecules that compose the chromatin milieu (3,5). The concept of chromatin damage emerged to summarize the accumulating evidence that implicates DNA intercalators in chromatin destabilization in the absence of DNA damage. Chromatin damage can result from various insults such as exposure to DNA intercalating agents or disturbances in chromatin structure and organization. A root cause of chromatin damage could be histone eviction (6). Understanding the causes and consequences of chromatin damage is important for studying the mechanisms of genome maintenance, cellular response to DNA damage, and the development of potential novel therapeutic strategies targeting cancer.

In recent years, progress has been made in the understanding of how DNA intercalators are capable of damaging the chromatin in the absence of DNA breaks. The anthracycline Aclarubicin is a DNA intercalator with a similar structure to the Topoisomerase II (Topo II) poison Doxorubicin but lacks the structure necessary to poison the enzyme and therefore is not capable of inducing Topo II-dependent DNA damage (7). At higher concentrations, Aclarubicin induces core histone eviction from open chromosome areas without inducing DNA double-strand breaks (8). Another interesting example is the small molecule CBL0137, a member of the curaxin family of carbazole-based DNA intercalators. Importantly, CBL0137 does not induce DNA damage (9), but it alters the shape of the DNA helix, which increases the inter-base-pair distance, and leads to the unwrapping of DNA from the histone octamer and to nucleosome destabilization (3,10). CBL0137 causes binding of FACT (FAcilitates Chromatin Transcription) to different components of disassembled chromatin in CBL0137-treated cells, a phenomenon described as chromatin trapping. FACT is a histone chaperone that participates in transcription and seems to prevent nucleosome loss during chromatin destabilization (3). Aclarubicin also induces chromatin trapping of FACT (5).

Quite a few of the DNA intercalating agents are also inhibitors of transcription. Actinomycin D is one of the best-studied ribosomal RNA (rRNA) synthesis inhibitors. It is also capable of inhibiting RNA Polymerase II (Pol II) activity at higher concentrations than those required to inhibit RNA Polymerase I (Pol I) and induce DNA damage possibly by interference with topoisomerase functions or DNA replication, reviewed in refs (11,12). Over the years several other DNA intercalators have been developed with a focus on targeting transcription, as both Pol I (ribosome biogenesis) and Pol II are attractive targets in anti-cancer therapy (13,14). Some DNA intercalating agents have a higher preference for the GC-rich nucleolar rDNA. BMH-21 is an acridine-like quinazolinone described as a DNA intercalator that blocks Pol I transcription, and it does not induce DNA damage (15,16). BMH-21 inhibits various stages of Pol I transcription, including initiation, promoter escape and elongation (17). Following BMH-21 treatment a reduction in Pol I occupancy and an increase in sequence-specific pausing upstream of GC-rich rDNA sequences was seen. BMH-21 is capable of triggering the degradation of largest catalytic subunit of the Pol I, POLR1A (RPA1) (15). The latter property is shared with Aminacrine, Ethacridine and two aminoquinolines; Amodiaquine and Amopyroquine (11). The capacity to induce degradation of POLR1A has been proposed as a relevant cytotoxic mechanism of BMH-21 (18). Our previous studies using BMH-21 confirmed that it is a potent inhibitor of Pol I, ribosome biogenesis and induces cell death. Notably, analysis of gene expression in BMH-21-treated cells indicated an unexpectedly great number of genes with perturbed expression, suggesting effects and targets beyond the inhibition of Pol I (19,20). Hence, it is necessary to better understand the mechanism of action of BMH-21 and how transcription is affected.

Despite belonging to three different compound classes, BMH-21, CBL0137 and Aclarubicin share overlapping functional features including DNA intercalation, induction of p53 and the inability to cause DNA damage. Here we demonstrate that BMH-21, Aclarubicin and CBL0137 induce degradation of Pol II, the chromatin trapping of FACT, TOP2A and TOP2B and drive the formation of Z-DNA. Furthermore, Aclarubicin and CBL0137, similar to BMH-21, are acting as Pol I inhibitors triggering nucleolar stress with implications for understanding the action of these compounds as anti-cancer treatments. In addition, BMH-21 mimics a catalytic inhibitor of Topoisomerase II, resembling the effects described for Aclarubicin. Finally, we show that siRNA-mediated knocking down of POLR2A (RPB1) induces a faster and more dramatic cytotoxic effect compared to the knocking down of POLR1A and POLR3A (RPC1). Taken together, our results show that BMH-21, Aclarubicin and CBL0137 share a larger effect on Pol II than previously thought and are impacting chromatin through several potentially cytotoxic effects simultaneously.

## Materials and methods

### Cell culture

Cell lines and culture conditions are described in Supplementary Table S1. Briefly, cells were reseeded 2–3 times per week. Tests for mycoplasma detection were performed monthly. All cell lines were maintained in DMEM with GlutaMAX (Gibco) supplemented with 10% fetal bovine serum and Pen/Strep.

### Antibodies and chemicals

Antibodies and the dilutions used in this study are listed in Supplementary Table S2. Chemicals are listed on the Supplementary Table S3. Compounds were dissolved in dimethyl sulfoxide (DMSO) or water according to the vendor's instructions. Stock dilutions were aliquoted and stored at −20°C. UV irradiation was performed using a CL-1000 UVP Ultraviolet Crosslinker.

### Immunoblotting

Sub-confluent cells were directly lysed in RIPA buffer (Thermo, PI-89901) plus protease (cOmplete ULTRA, code 05892970001, Roche) and phosphatase inhibitors (PhosSTOP, code 04906837001 Roche) and sonicated during 7 cycles of 30 s on and 30 s off, in a Bioruptor® (Diogenode). Protein concentration was quantified with DC™ Protein Assay Kit II (Bio-Rad, 5000112). Two to 10 μg were boiled in Laemmli sample buffer for 5 min at 95°C, loaded onto SDS-PAGE gels and transferred to nitrocellulose or PVDF membranes. Chemiluminiscence signal was detected using SuperSignal™ West Dura (Thermo, 34076). Images were acquired with Amersham Imager 680 scanner. The Pierce Subcellular Protein Fractionation Kit (78840) was used for the extraction of chromatin-bound, nuclear soluble and cytoplasmic fractions, following the manufacturer's instructions. Immunoblot analysis was performed a minimum of two times with independent biological replicates. Immunoblot quantification was performed in Image Lab (Biorad), normalizing protein signal with the corresponding loading control. Values under immunoblots represent ratios of protein levels compared to their respective controls and normalized against the loading control.

### siRNA knockdown and viability assay

Cells were transfected with 20 nM siRNA using Lipofectamine™ RNAiMAX during 4h in Opti-MEM (Gibco, 31985070) and further incubated overnight in suitable complete cell media. ON-TARGET plus non-targeting pool (D-001810-10-20) was used as siRNA control (siControl). The following siRNA Flexitube oligos were purchased from Qiagen: Hs_POLR1A_5 (siPOLR1A #1), Hs_POLR1A_6 (siPOLR1A #2), Hs_POLR2A_5 (siPOLR2A #1), Hs_POLR2A_6 (siPOLR2A #2), Hs_POLR3A_7 (siPOLR3A #1) and Hs_POLR3A_8 (siPOLR3A #2). For viability assessment, cells were seeded in 96 well-plates in triplicate treatments and treated as indicated. Viability was assessed using the CellTiter-Glo® Luminescent Cell Viability Assay (Promega) following manufacturer's instructions and measured in the Tecan Infinite M1000Pro plate reader. The siControl samples for every timepoint and cell line were used as controls, and all data were calculated as a percentage of their respective controls.

### Immunofluorescence and imaging

Cells grown in 96-well imaging plates were fixed in 4% formaldehyde for 15 min at room temperature, washed with PBS three times, permeabilized in PBS 0.5% Triton X-100 for 10 min and blocked with PBS 3% bovine serum albumin for 30 min. Cells were sequentially incubated with the primary (overnight) and secondary antibody (2 h), and stained with Hoechst 2 μM for 15 min. Images were acquired using an INCell Analyzer 2200 (GE Healthcare) or a Nikon Eclipse Ti2 inverted epifluorescence microscope. Image segmentation and signal quantification was performed using Cell Profiler software (21).

### RADAR assay

U2OS cells (0.8 × 10^6^) were washed once with PBS and lysed adding 1 ml of DNAzol (ThermoFisher CAT#10503027). Nucleic acids were precipitated by adding 0.5 ml of ethanol 100%, incubated for 5 min at –20°C and centrifugated at 12 000 × g for 10 min. Precipitates were washed twice in ethanol 75% and resuspended in 300 μl of 8 mM NaOH, heated at 65 °C for 15 min and sonicated at low intensity for 15 s ON and 15 s OFF, 5 times. Samples were centrifugated at 12 000 × g 10 min and the supernatant containing nucleic acids with covalently bound proteins were collected. Nucleic acids containing protein adducts were quantitated and 200 ng of nucleic acids were slot-blotted on a PVDF membrane (Immobilon-FL 0,45 μM pore size, Merck Millipore). Membranes were crosslinked with UV light. TOP2A, TOP2B and double-stranded DNA (ds-DNA) were detected as described in the immunoblotting section.

### Quantitative real-time reverse transcription PCR

Total RNA was extracted with PureLink™ RNA Mini Kit (ThermoFisher) following the manufacturer's instructions and qPCR was performed using Power SYBR® Green RNA-to-CT™ 1-Step Kit (4389986, ThermoFisher) in a QuantStudio 5 PCR System. Cycling parameters: Reverse transcription at 48°C for 30 min, initial denaturation at 95°C for 30 s and 40 cycles of 95°C for 15 s and 62°C for 60 s. Melt curve analysis: 95°C for 15 and a gradual increase in temperature to 95°C (0.075°C/s). Three biological samples and three technical replicates per sample were analysed. Relative quantity was analysed using the ΔΔCt method using ACTB and QARS mRNAs as internal normalizers. Primer sequences are listed in Supplementary Table S4. Primer sequences for 47S rRNA and its 18S 5′and 3′junctions were obtained from (22).

### Statistics and reproducibility

Statistical significance was determined using t-tests or ANOVA tests with GraphPad Prism. RT-qPCR data are shown as means ± SD; *n* = 3 biological replicates. Student's *t* and ANOVA tests were performed when suitable and stated in the corresponding figures along with statistical significances.

## Results

### BMH-21 triggers degradation of POLR2A

Pol II regulation largely depends on the carboxy-terminal domain of its largest subunit POLR2A (RPB1), which contains multiple repeats of a consensus heptamer sequence. This region is highly phosphorylated during Pol II transcription. To measure protein levels of POLR2A we used the antibody clone D8L4Y to detect both unphosphorylated (IIa) and hyperphosphorylated (IIo) states of POLR2A. This is important to take into consideration since it has been shown that UV-radiation (23) and Actinomycin D (24) can induce POLR2A hyperphosphorylation, involving an almost complete shift of the unphosphorylated IIa to the hyperphosphorylated state IIo (23). Following transcription initiation, the IIo form can be ubiquitinated and degraded when encountering DNA lesions (25). It has been proposed that this IIa → IIo → degradation cycle is repetitive until the DNA lesions are removed, and eventually the cell run out of free Pol II (IIa) (26). Therefore, relying exclusively on phosphorylated state (IIo) to measure POLR2A stability may lead to over- or underestimation of the actual cellular levels. Measuring IIa levels provides thus a more reliable assessment of Pol II degradation, in addition to its phosphorylated states.

**Figure 1.**
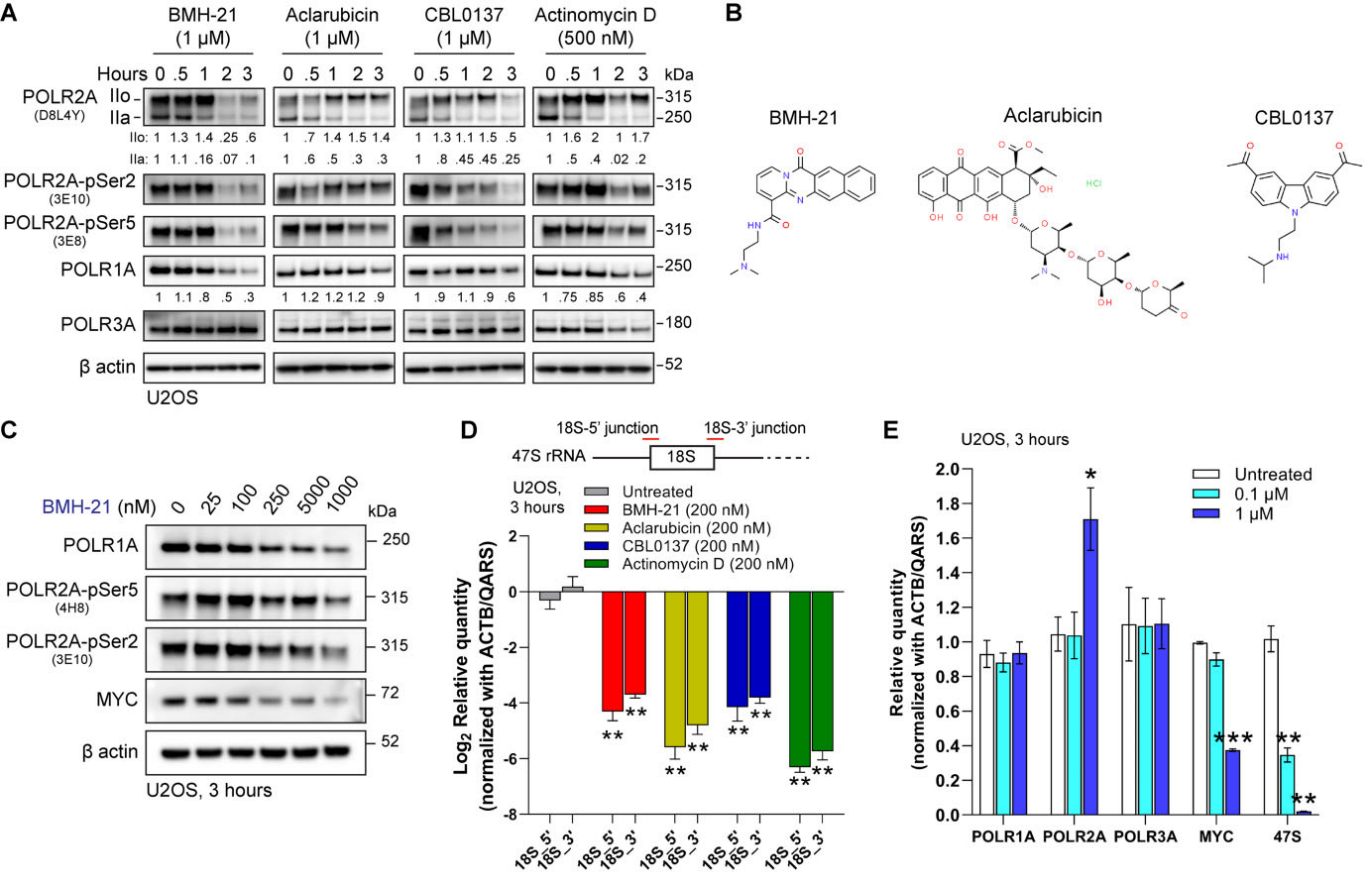
DNA intercalators induce the degradation of POLR2A. (**A**) Immunoblot analysis of total POLR2A (D8L4Y), POLR2A-pSer5 (3E8) POLR2A-pSer2 (3E10), POLR1A and POLR3A forms in U2OS cells treated with 1 μM of BMH-21, Aclarubicin, CBL0137 and 500 nM Actinomycin D for 3 h. The figure is representative of three independent biological replicates, assessing the impact of the compounds on POLR2A and POLR1A levels. Values under immunoblots represent ratios of POLR2A IIo, IIa and POLR1A levels compared to timepoint 0 and normalized using their respective β actin signal. (**B**) Chemical structures of BMH-21, Aclarubicin and CBL0137. (**C**) Immunoblot analysis of U2OS cells treated with increasing concentrations of BMH-21 for three hours. The blots shown are representative of more than three independent experimental reproductions. (**D**) RT-qPCR analysis of 18S 5′and 3′junctions of the *47S rRNA*, in cells treated with 200 nM of BMH-21, Aclarubicin, CBL0137 and Actinomycin D for 3 h. Data are presented as the mean ± SD (*n* = 3), **P value < 0.01 by Student's *t* test versus untreated cells (**E**) RT-qPCR analysis of *POLR1A*, *POLR2A*, *POLR3A*, *MYC* and *47S* rRNA. Data are presented as the mean ± SD (*n* = 3), *P value < 0.05, **P value < 0.01, ***P value < 0.001 by ANOVA test versus untreated cells.

To our surprise, we found that 1 μM of BMH-21, Aclarubicin, CBL0137 and 500 nM of Actinomycin D induced a time dependent degradation of POLR2A, with a progressive consumption of form IIa (Figure 1A). This correlated with the reduction of phosphorylation in Serine 5 (Ser5) and Serine 2 (Ser2) of POLR2A (Figure 1A), associated with initiation and productive elongation, respectively (27). Levels of POLR3A (RPC1), the catalytic subunit of RNA Polymerase III (Pol III), were affected only at later timepoints in the case of Actinomycin D. Taken together, this data shows that these DNA intercalators induce a reduction of total levels of POLR2A, especially BMH-21, for which POLR2A follows a similar degradation trend in U2OS cells as its previously described effect on POLR1A (15). Figure 1B depicts the chemical structures of BMH-21, Aclarubicin and CBL0137.

Interested in BMH-21′s effect on POLR2A, we tested a broader panel of chemotherapeutic as well as experimental compounds. We observed that CX-5461 and Mitoxantrone, both DNA intercalators and Topo II poisons (28–30), induced a reduction of POLR2A levels (Supplemental Figure S1A). As expected, the effect of Triptolide and 4-NQO on Pol II is similar to those described elsewhere (26,31). Levels of POLR1A were affected by BMH-21 and CX-5461 and to a lesser extent by Doxorubicin and Mitoxantrone (Supplemental Figure S1A). We confirmed the reduction of POLR1A in U2OS cells treated with a high concentration of CX-5461, but not in BJ fibroblasts (Supplementary Figure S1B), a cell type where POLR1A degradation is not observed upon treatment with DNA intercalators such as Amodiaquine (19). In addition, BMH-21 induces a decrease in the level of the transcription factor MYC (Figure 1C), just as Actinomycin D, Mitoxantrone, Triptolide and 4-NQO (Supplementary Figure S1). Of notice, CBL0137 has previously been described as capable of triggering the degradation of MYC (10). Taken together, POLR2A degradation is induced by BMH-21 and other DNA intercalators.

Actinomycin D is a DNA intercalator known to inhibit Pol I transcription at low concentrations (5–50 nM), but at high concentrations is also capable of inhibiting Pol II (100–500 nM) (11). We used Actinomycin D as a control given its wide application as an RNA polymerase inhibitor, however, we observed differences when it was compared to BMH-21, Aclarubicin and CBL0137. Actinomycin D induces phosphorylation of Ser139-H2AX (yH2AX), a marker of DNA damage (Supplementary Figure S1A). Induction of yH2AX by Actinomycin D has been described previously (32) and it is probably related to its capacity to stabilize Topo I-DNA covalent complexes, therefore inducing DNA damage (33). However, yH2AX induction has not been described for BMH-21 (16), Aclarubicin (34) or CBL0137 (9). In addition, Actinomycin D induces a hyperphosphorylation of form IIo and increases in Ser2 and Ser5 (Figure 1A, Supplementary Figure S1A), in agreement with previously published data (24). Although Actinomycin D also induces degradation of Pol II (Figure 1A) as BMH-21, Aclarubicin and CBL0137, these compounds do not seem to induce strong changes in Pol II phosphorylation states as Actinomycin D does, under the conditions described here. Nonetheless, we included Actinomycin D because it is a DNA intercalator and Pol I inhibitor, however, it also triggers DNA damage and induces Pol II phosphorylation, hence, despite similarities with the effects of BMH-21, Aclarubicin and CBL0137 described here, caution should be taken in consideration when drawing conclusions for Actinomycin D.

Both BMH-21 and Actinomycin D are established Pol I inhibitors. Here we show that Aclarubicin and CBL0137 also significantly inhibited the synthesis of the primary rRNA transcript *47S*, using primers targeting the junction between 18S sequence and 5′ETS (18S-5′junction) and ITS1 (18S-3′junction) regions (Figure 1D). Next, we aimed to compare the effects of CBL0137, Aclarubicin, BMH-21 and Actinomycin D on the nucleolar morphology and rDNA transcription in U2OS cells. The impact of these treatments on nucleolar morphology was assessed using immunofluorescence for nucleolar proteins fibrillarin (FBL) and nucleophosmin 1 (NPM1) (Supplementary Figure S2A). All four compounds disrupted nucleolar morphology, causing translocation of NPM1 to the nucleoplasm and nucleolar shrinkage (Supplementary Figure S2A). To further confirm the disruptive effect of Aclarubicin in the nucleolus, double immunostaining for POLR1A and 5.8S rRNA was performed in U2OS and BJ fibroblasts at concentrations of 0.1 and 1 μM. This revealed a notable reduction in nucleolar 5.8S rRNA staining (Supplementary Figure S2B and S2C). Our findings demonstrate that BMH-21, Aclarubicin and CBL0137 effectively inhibit Pol I transcription and disrupt nucleolar morphology. This effect on the nucleolus is likely to contribute to the p53 stabilization through the impaired ribosomal biogenesis checkpoint involving ribosomal proteins RPL5 and RPL11 (35) in the case of the three compounds.

In cells treated with BMH-21 we observed an up-regulation of *POLR2A* mRNA in the U2OS cell line, with a simultaneous down-regulation of the *MYC* mRNA and *47S* rRNA levels (Figure 1E). This effect was also observed in BJ cells, but it was not statistically significant in HT29 and RKO cell lines (Supplementary Figure S1C). Furthermore, we analyzed the effect of BMH-21, Aclarubicin and CBL0137 on the mRNA levels of all twelve Pol II subunits. A statistically significant increase was observed only for *POLR2A* mRNA under BMH-21 and CBL0137, but not for Aclarubicin (Supplementary Figure S1D). This paradoxical expression of *POLR2A* mRNA under conditions that will lead to POLR2A degradation can be partially explained by recent findings in chromatin dynamics observed for CBL0137 and Aclarubicin. A study found that genes activated by CBL0137 had the highest index in Pol II paucity under basal conditions, accompanied by high levels of activating histone markers. This indicate either a higher sensitivity for transcriptional activation in these genes, or the presence of additional mechanisms for the release of paused Pol II (9). In addition, Aclarubicin is capable of increasing levels of elongating Pol II in gene bodies upon treatment with 1 μM for 30 min, indicating that Aclarubicin as well targets the conversion of initiating Pol II into the elongating form (36). Thus, rapid Pol II transcription can take place upon treatment with DNA intercalators, therefore indirectly supporting our finding that *POLR2A* mRNA expression is induced upon treatment with BMH-21 and CBL0137, in conditions that ultimately will conclude with the degradation of POLR2A.

### Degradation of POLR2A by BMH-21 is dependent on p97, ubiquitin ligase and proteasome activity

Next, we compared the dependency of POLR1A and POLR2A degradation on proteostatic factors involved in protein degradation, including: i) the ubiquitin-dependent protein segregase p97, using the inhibitor CB-5083; ii) the requirement of a Cullin-RING ubiquitin ligase, using the NEDDylation inhibitor MLN-4924, that prevents the conjugation of the ubiquitin-like protein NEDD8 to activate Cullin-RING ubiquitin ligases; and iii) the proteasome inhibitor MG-132.

**Figure 2.**
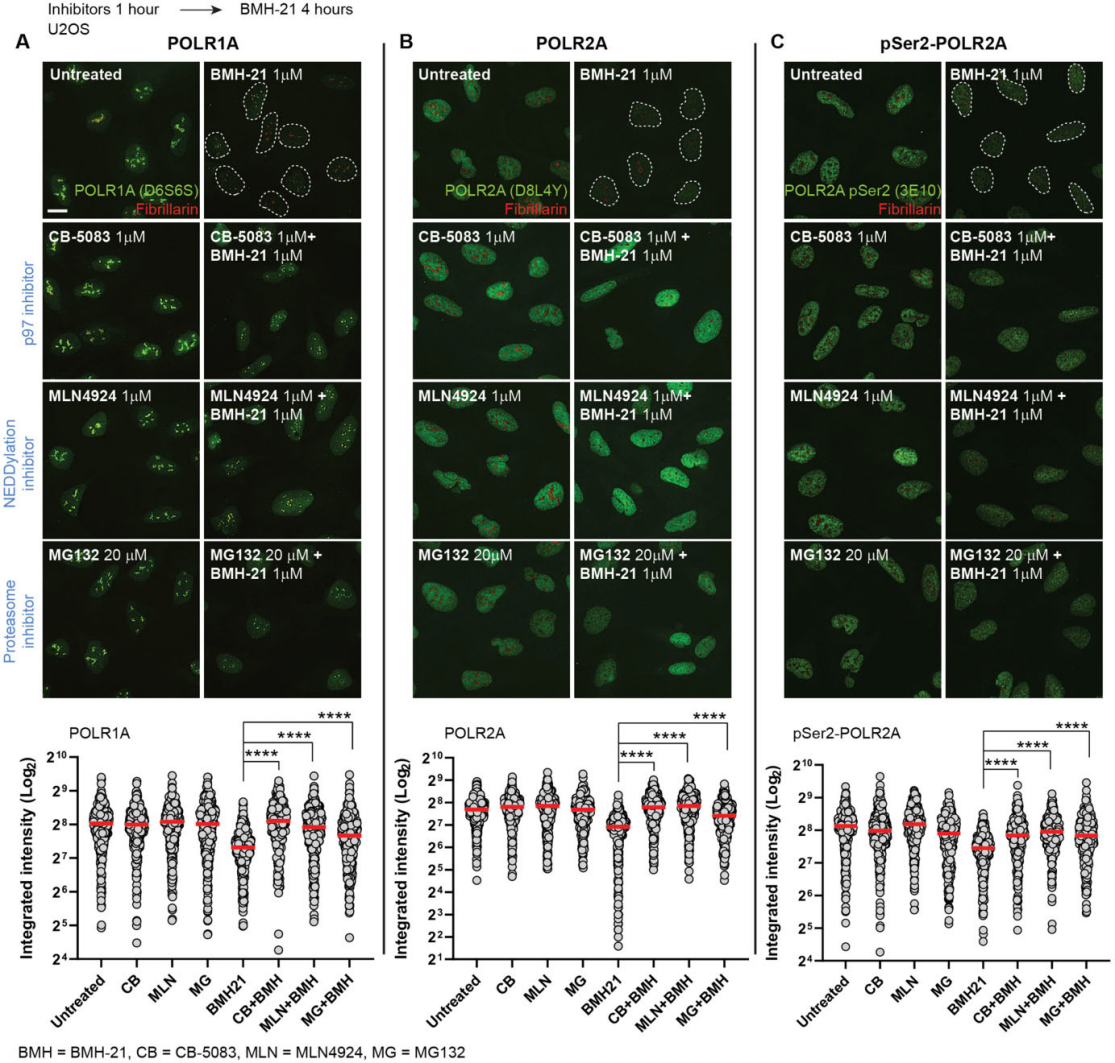
POLR2A degradation is dependent on p97, ubiquitin ligase and the proteasome activity. Representative images and signal quantification of immunofluorescence of (**A**) POLR1A, (**B**) POLR2A (D8L4Y) and (**C**) pSer2-POLR2A (3E10) (green channel) and Fibrillarin (red channel) in U2OS cells treated with BMH-21 (1 μM) and p97 inhibitor (CB-5083, 1 μM), NEDDylation inhibitor (MLN4924, 1 μM) and proteasome inhibitor (MG132, 20 μM). Scale bar 10 μm. Cells were pre-incubated with inhibitors for 1 hour and then co-incubated with BMH-21 for 4 additional hours. Image quantification of POLR1A, POLR2A (D8L4Y) and POLR2A-pSer (3E10) was analyzed based on integrated intensity (arbitrary units) (*n* > 300 cells per condition) by one-way ANOVA test, ****P value < 0.0001. Red bars represent the mean.

Upon BMH-21 treatment, the degradation of POLR1A is prevented by the inhibitors CB-5083, MLN4924 and MG132 (Figure 2A), indicating dependency on p97, a Cullin-RING ubiquitin ligase and the proteasome. Previously, it has been shown that POLR1A degradation by BMH-21 required a Cullin-RING ligase (37) and the proteasome (15). Here, we show that BMH-21-triggered POLR2A degradation (Figure 2B) and pSer2-POLR2A degradation (Figure 2C) are both rescued by CB-5083, MLN4924 and MG132, indicating that POLR2A is also dependent on p97, a Cullin-RING ubiquitin ligase and the proteasome activity. Taken together, degradation of both Pol I and Pol Il catalytic subunits by BMH-21 have in common a similar proteostatic pathway.

### Positioning of pol II on chromatin is required for its degradation induced by DNA intercalators

Aclarubicin and CBL0137 are examples of compounds other than BMH-21 that intercalate DNA, do not induce DNA damage, and yet activate p53. CBL0137 alters the topology of the DNA helix without inducing DNA breaks. It blocks topoisomerase activity leading to super-helical stress and potentially requiring base unpairing to form alternative DNA structures (10,38). Aclarubicin is a strong DNA intercalator that inhibits Topo II and does not induce DNA breaks (6). BMH-21 is thought to unwind DNA helix by stacking flat between GC-bases (18). We showed that BMH-21, Aclarubicin and CBL0137 induce degradation of Pol IIa (Figure 1A). Since the phosphorylation states of Pol II correlate with its chromatin positioning and activity, we sought to study how the phosphorylation states relate to this compound-induced degradation.

**Figure 3.**
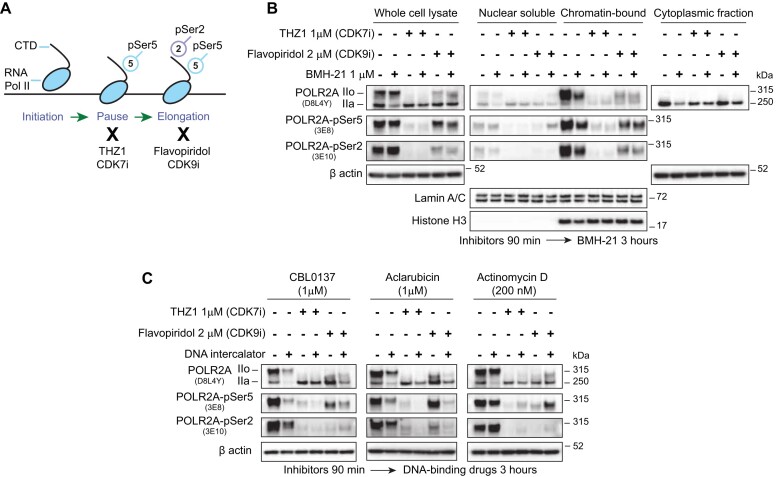
Inhibition of Pol II by CDKis prevents its degradation. (**A**) Schematic representation of Pol II transcription cycle and effect of the transcription inhibitors. THZ1 inhibits the phosphorylation of Ser5 by Cdk7. Flavopiridol inhibits the phosphorylation of Ser2 by Cdk9. (**B**) Immunoblot of whole-cell and subcellular fractions of U2OS cells treated with CDK inhibitors THZ1 and Flavopiridol for 90 min and then co-incubated with BMH-21 1 μM for 3 additional hours. (**C**) Immunoblot analysis of whole-cell lysates using of U2OS cells treated with CDK inhibitors as in (**B**) and co-incubated with CBL0137 (1 μM), Aclarubicin (1 μM) and Actinomycin D (200 nM).

During the initiation of Pol II transcription, the CDK7 subunit of TFIIH phosphorylates the Ser5 of the POLR2A CTD, establishing the promoter-proximal pause in Pol II elongation, downstream of the transcription start site (27). Subsequently, CDK7 will phosphorylate the CDK9 subunit of P-TEF-b to induce a release of Pol II into productive elongation, phosphorylating POLR2A CTD on Ser2 and factors DSIF and NELF (27,39) (Figure 3A). BMH-21 induced a reduction of both Ser5 and Ser2 phosphorylation of POLR2A (Figure 1A), therefore, we explored if chromatin positioning is necessary for POLR2A degradation. We treated cells with the CDK7 inhibitor THZ1 and the CDK9 inhibitor Flavopiridol (Figure 3A and B). THZ1 treatment resulted in the disappearance of hyperphosphorylated POLR2A (IIo), while not affecting levels of the IIa form, as evidenced by the loss of Ser5 and Ser2 phosphorylation states from the chromatin-bound fractions (Figure 3B). Flavopiridol treatment also induced a decrease of IIo but did not affect IIa levels, with a decrease in phosphorylated Ser2, and an expected retention of partially phosphorylated POLR2A-Ser5. Of notice, both phosphorylated states (IIo) are preferentially enriched on chromatin fractions, while IIa is preferentially found at the nucleoplasm and cytoplasmatic fractions (Figure 3B).

Treatment with both CDK7 and CDK9 inhibitors partially or completely rescued the degradation of IIa induced by BMH-21, indicating that chromatin positioning is required for degradation of the enzyme, even affecting the levels of IIa present in the cytoplasm (Figure 3B). A similar trend was seen in cells treated with CBL0137, Aclarubicin or Actinomycin D (Figure 3C). Pol II already positioned on chromatin, as in the case of Flavopiridol-treated cells, where a remaining amount of Ser5-Pol IIo is still present in chromatin fractions, may still be susceptible to degradation, as seen for BMH-21, CBL0137 and Aclarubicin (Figure 3B and C). Actinomycin D induces hyperphosphorylation of Pol II, likely related to the DNA damage induced at higher concentrations (Supplementary Figure S1A). This may explain the mild increase in Pol II Ser2 and Ser5 seen even after treatment with CDK7i and CDK9i (Figure 3C). However, we still observed Pol II degradation that is partially rescued by CDK7i and CDK9i (Figure 3C).

### DNA intercalators modify the localization of chromatin proteins

To better understand how these four compounds impact chromatin, we measured their effect on several relevant chromatin factors, including both forms of Topo II (TOP2A and TOP2B), Topo I (TOP1), both subunits of the histone chaperone FACT (SPT16 and SSRP1) and the catalytic subunits of RNA Pols I, II and III (POLR1A, POLR2A and POLR3A, respectively).

**Figure 4.**
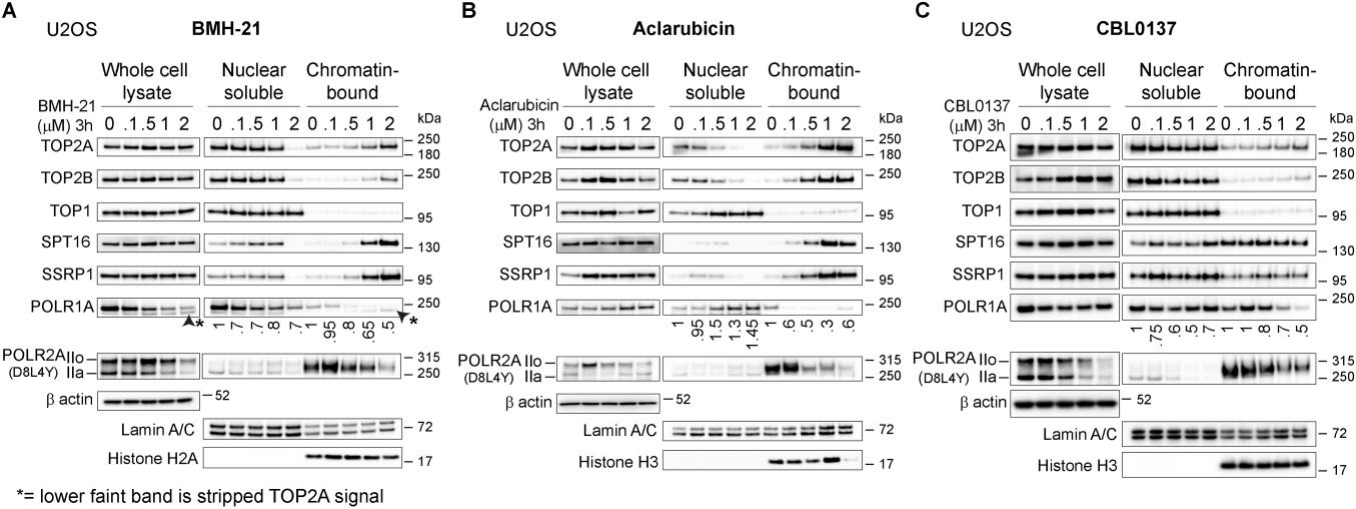
DNA intercalators induce chromatin trapping and detachment of chromatin factors and enzymes. Immunoblot analysis of U2OS whole cell lysates and subcellular fractions treated with increasing concentrations of (**A**) BMH-21, (**B**) Aclarubicin and (**C**) CBL0137. Values under immunoblots represent ratios of POLR1A levels compared to concentration 0 and normalized using their respective Lamin A/C signal.

BMH-21 induces degradation of POLR1A in several cancer cell lines including U2OS, but this degradation is not universal, and therefore is not observed in some cancer and normal-like cell lines (15), including BJ fibroblasts (19). Surprisingly, we observed that in BJ fibroblast, BMH-21 induces POLR2A degradation in the absence of POLR1A degradation (Supplementary Figure S3A and S3B). Moreover, BMH-21 induces loss of POLR1A from chromatin in both U2OS and BJ cells (Figure 4A and Supplementary Figure S3B). In addition, BMH-21 induces Pol IIa degradation in other cancer cell lines (Supplementary Figure S3A).

Aclarubicin, CBL0137, and Actinomycin D each trigger loss of POLR1A from chromatin while in contrast to BMH-21 there was no decrease in the nuclear soluble fractions in the case of these drugs (Figure 4B, C and Supplementary Figure S3C–E), which is compatible with the inhibition of Pol I transcription observed in Figure 1D. In agreement with Figure 1A, we observed that BMH21, Aclarubicin, CBL0137 and Actinomycin D induced a concentration-dependent degradation of both Pol II forms, IIo and IIa, upon increasing concentrations (Figure 4A–C and Supplementary Figure S3B–E).

CBL0137 and Aclarubicin are known for inducing the chromatin trapping of SSRP1 and SPT16, subunits of the histone chaperone FACT (5). We found that BMH-21also can induce trapping of FACT at similar concentrations as Aclarubicin (Figure 4A–C & Supplementary Figure S3B and C). In our conditions, the described trapping effect of CBL0137 as previously described in for example HeLa cells (38), was not evident in U2OS cells and only modest in BJ cells (Figure 4C and Supplementary Figure S3D). Intriguingly, TOP2A and TOP2B are retained on chromatin fraction upon increasing concentrations of BMH-21, Aclarubicin and CBL0137, resembling the pattern observed for FACT subunits (Figure 4A–C and Supplementary Figure S3B–D). On the contrary, Topoisomerase I (TOP1) is not retained on chromatin upon drug treatment (Figure 4A–C and Supplementary Figure S3B–D).

### BMH-21, Aclarubicin and CBL0137 are histone evictors

**Figure 5.**
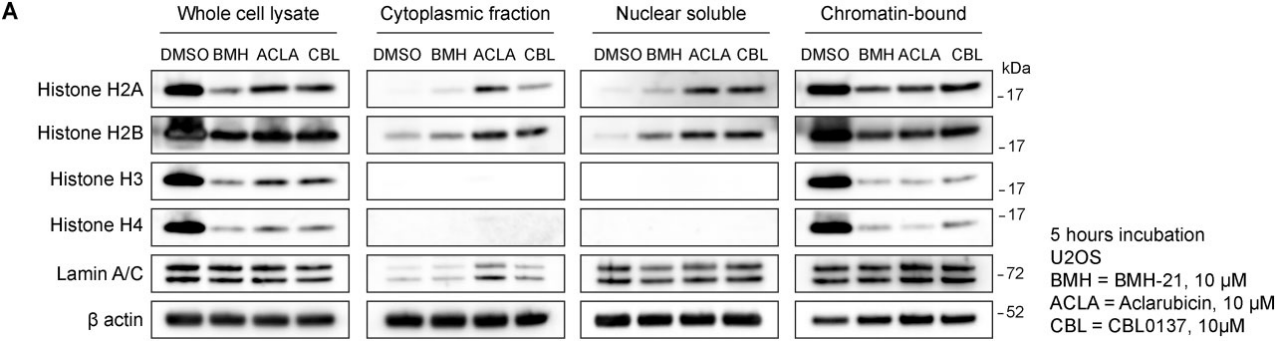
BMH-21, Aclarubicin and CBL0137 trigger the eviction of core histones. (**A**) Immunoblot analysis of core histones in whole cell lysate and cellular fractionations of U2OS cells treated with BMH-21 (10 μM), Aclarubicin (10 μM) and CBL0137 (10 μM) for 5 h.

Aclarubicin has been described as a histone evictor, capable of inducing the removal of histones from chromatin, albeit at high concentrations (>10–20 μM) (6,34). In a similar fashion, CBL0137 can also induce loss of histones (38,40). In chromatin-bound fractions, we observed fluctuating levels of Histone H3 following Aclarubicin treatment (Figure 4B and Supplementary Figure S3C), and a similar trend was observed for histone H2A upon BMH-21 treatment (Figure 4A and Supplementary Figure S3B). Histone protein levels were originally assessed as loading control for chromatin fractions, however, due to their varying levels we decided to increase compound concentrations and study histone eviction for BMH-21, Aclarubicin and CBL0137. Upon treatment with 10 μM for 5 h we detected a clear pattern of histone eviction and depletion from chromatin fractions (Figure 5). Interestingly, core histones H2A and H2B were detected in nuclear soluble and cytoplasmatic fractions, contrary to core histones H3 and H4, that disappeared quickly from nuclear and chromatin fractions (Figure 5).

BMH-21, Aclarubicin and CBL0137 do not affect the levels of POLR3A (Figure 1A and Supplementary Figure S3F). However, these three compounds induce loss of POLR3A from chromatin at 5 μM for 3 h, and its accumulation in nuclear soluble fractions (Supplementary Figure S3F). Intriguingly, these compounds also affect the chromatin extractability of UBF (Upstream Binding Factor), a nucleolar factor specifically bound to active rDNA repeats and involved in the formation of active nucleolar chromatin (41) (Supplementary Figure S3F). Taken together, BMH-21, Aclarubicin, and CBL0137 trigger an array of similar effects, compromising the function of nuclear RNA Polymerases and inducing either trapping/recruitment or release and/or degradation of enzymes and chromatin factors, in a dose-dependent manner.

### BMH-21 acts as a catalytic inhibitor of Topo II

DNA Topoisomerases are relevant enzymes that solve DNA topological problems during replication and transcription (42). Both Topo II isoforms, TOP2A and TOP2B, can relax negatively supercoiled DNA and carry out DNA decatenation. Compounds targeting Topo II are divided into two classes: Topo II poisons and Topo II catalytic inhibitors. Topoisomerase poisoning takes place when the enzyme cuts DNA but is not capable of re-ligate, generating a DNA-Topo cleavage complex that leads to activation of the DNA damage response. This process can be induced by drugs such as the Topo I poisons Camptothecin and Topotecan, as well as the Topo II poisons Doxorubicin, Etoposide and Mitoxantrone (30). On the other hand, Topo II catalytic inhibitors inhibit both its enzymatic activities, cutting and re-ligation, therefore they do not generate Topo II covalent complexes or DNA damage. The most relevant ones are the bisdioxopiperazines, such as ICRF-187, that inhibit non-competitively Topo II ATPase activity and trap Topo II as a closed clamp (43).

**Figure 6.**
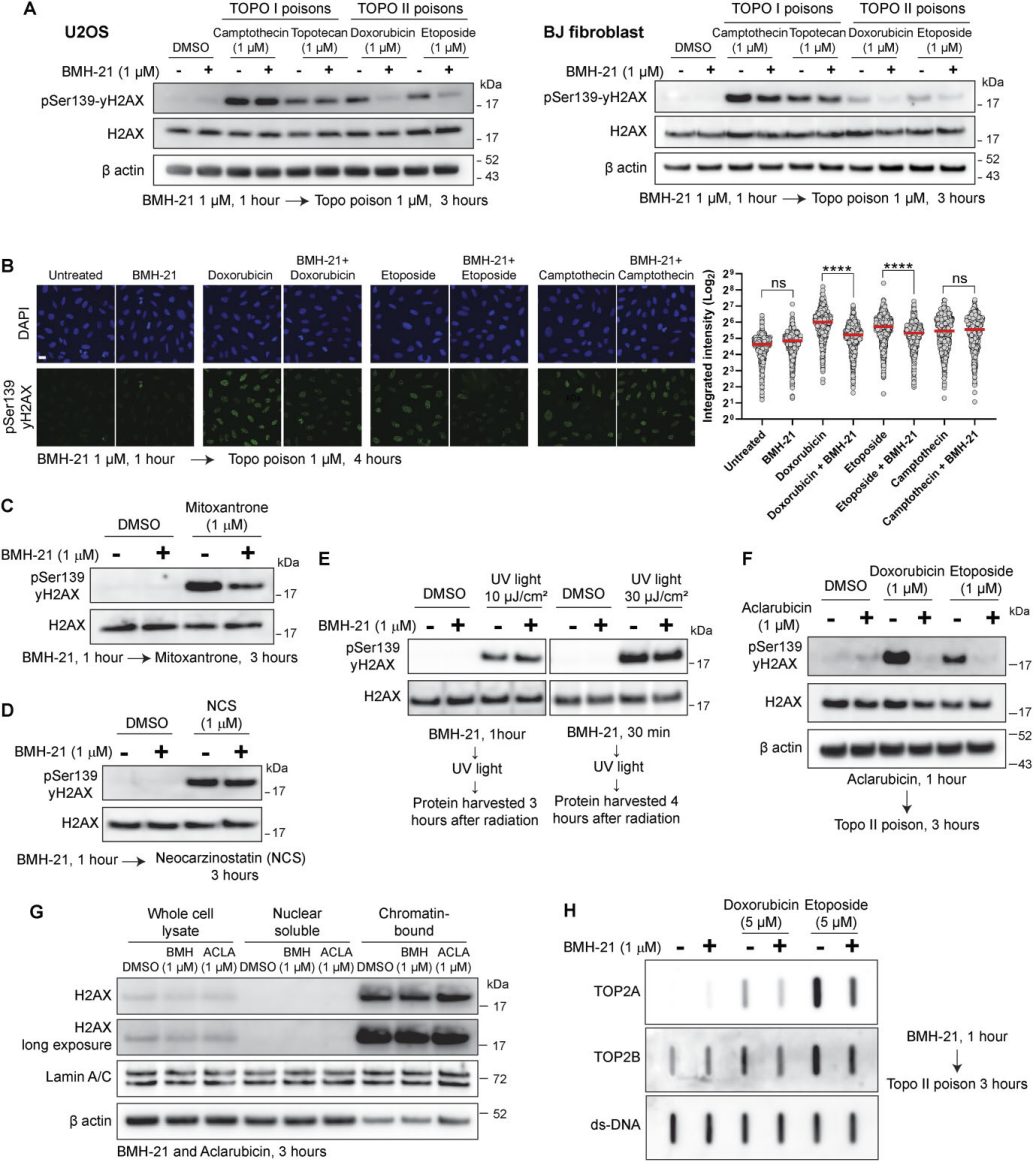
BMH-21 acts as a catalytic inhibitor of Topo II. (**A**) Immunoblot analysis of phophoSer139-H2AX in U2OS and BJ cells pre-treated with BMH-21, in the presence and absence of Topo I and Topo II poisons for 3 hours. (**B**) Representative images and immunofluorescence quantification of integrated intensity (arbitrary units) of phophoSer139-H2AX signal in cells pretreated with BMH-21 for 1 hour and subsequently treated with Doxorubicin, Etoposide and Camptothecin for an additional 4 hours. Scale bar 10 μm. Data was analyzed (*n* > 800 cells per condition) by one-way ANOVA test, ns = non-significant, ****P value < 0.0001. Red bars represent the mean. (**C**) Immunoblot analysis of phophoSer139-H2AX in U2OS cells treated with Topo II poison Mitoxantrone after pre-treatment with BMH-21. (**D**) Immunoblot analysis of phophoSer139-H2AX in U2OS cells treated with Neocarzinostatin after pre-treatment with BMH-21. (**E**) Immunoblot analysis of phophoSer139-H2AX in U2OS cells treated with two doses of UV light after pre-treatment with BMH-21. (**F**) Immunoblot analysis of phophoSer139-H2AX in cells treated with Doxorubicin and Etoposide for 3 hours after pre-treatment with Aclarubicin for 1 h. (**G**) Immunoblot analysis of H2AX in U2OS whole cell lysate and cellular fractionations after treatment with BMH-21 (1 μM) and Aclarubicin (1 μM). (**H**) RADAR assay for detection of TOP2Acc and TOP2Bcc in U2OS cells pre-treated with BMH-21 (1 μM), followed by treatment with Doxorubicin (5 μM) and Etoposide (5 μM) for 3 additional hours. Equal loading was determined by probing with an anti-double stranded DNA antibody (ds-DNA). The figure is representative of two independent experiments.

Intrigued by the chromatin trapping observed for TOP2A and TOP2B in cells treated with BMH-21, Aclarubicin and CBL0137 (Figure 4A-C), we analyzed the effect of BMH-21 on Topoisomerase activity. Pre-treating cells with BMH-21 prevented phosphorylation of Ser139-H2AX (yH2AX), induced preferentially by Topo II poisons but not by Topo I poisons, assessed via immunoblots (Figure 6A) and immunofluorescence microscopy image analysis (Figure 6B). In addition, BMH-21 reduced the yH2AX induced by Topo II poison Mitoxantrone (Figure 6C), but no reduction was seen under the radiomimetic Neocarzinostatin (Figure 6D) or UV radiation (Figure 6F), suggesting that BMH-21 is interfering preferentially with Topo II activity, but does not interfere with other sources of DNA damage. Interestingly, this resembles the findings observed for Aclarubicin, an anthracycline structurally similar to the Topo II poison Doxorubicin, but lacks the capacity to poison Topo II, while retaining its capacity to intercalate DNA (7). Intriguingly, Aclarubicin has been classified as a Topo II catalytic inhibitor, because it is capable of preventing the DNA damage induced by Topo II poisons such as Etoposide (44), therefore mimicking the inhibition of Topo II activity induced by Topo II catalytic inhibitors (43). Indeed, we observed that Aclarubicin is preventing the yH2AX induced by Doxorubicin and Etoposide (Figure 6F), in similar concentrations and timepoints we observed for BMH-21 (Figure 6A and B). Lower concentrations of BMH-21 also interfere with Topo II poisoning (Supplementary Figure S4A).

It has been shown that the eviction of H2AX variant may lead to an attenuation of the DNA damage response to DNA strand breaks (6). We have shown that at high concentrations (10 μM), BMH-21, Aclarubicin and CBL0137 induce eviction of Histone H2A (Figure 5), therefore we evaluated if the attenuation of the yH2AX signal we were observing was a consequence of eviction of variant H2AX. However, we did not observe eviction of H2AX for 1 μM of BMH-21 or Aclarubicin for 3 h (Figure 6G).

DNA-Topo II cleavage complex (TOP2cc) is an intermediate covalent state during Topo II catalytic cycle. Although TOP2ccs are readily reversible in normal conditions, they become stable under the effect of Topo II poisons (42). These lesions can be detected with RADAR (rapid approach to DNA adduct recovery) assays, that isolate nucleic acids with covalently bound protein adducts (45). We could observe that BMH-21 decreased the levels of TOP2Acc and TOP2Bcc upon treatment with Doxorubicin and Etoposide (Figure 6H), in agreement with the reduction of yH2AX seen in Figures 6A and B.

Since Topo II is required during transcription to manage the DNA supercoiling generated ahead and behind transcription machinery, we hypothesized that a reduction in transcription may result in an apparent reduction of Topo II activity, and therefore explain a reduction in Topo II-dependent DNA damage. We treated cells with transcription inhibitor Actinomycin D and Triptolide, an inhibitor of TFIIH and therefore an inhibitor of Pol II transcription initiation (46). We observed that Actinomycin D and Triptolide do not prevent the yH2AX induced by Doxorubicin and Etoposide (Supplementary Figure S4B), indicating that a general reduction in Pol II transcription, and therefore an expected decrease in transcription-associated Topo II activity, do not explain the prevention of yH2AX we observed for BMH-21 and Aclarubicin.

Collectively, these results show that BMH-21 also interferes with Top II activity, mimicking the effect of a Topo II catalytic inhibitor, similar to Aclarubicin. Although this may contribute to the cytotoxicity of BMH-21 and Aclarubicin, it also suggests that Topo II is being recruited to the chromatin to resolve topological stress, but its activity is compromised and is trapped on the chromatin upon treatment with a non-poison DNA-binding drug.

### BMH-21, Aclarubicin, CBL0137 and Actinomycin D induce formation of Z-DNA

**Figure 7.**
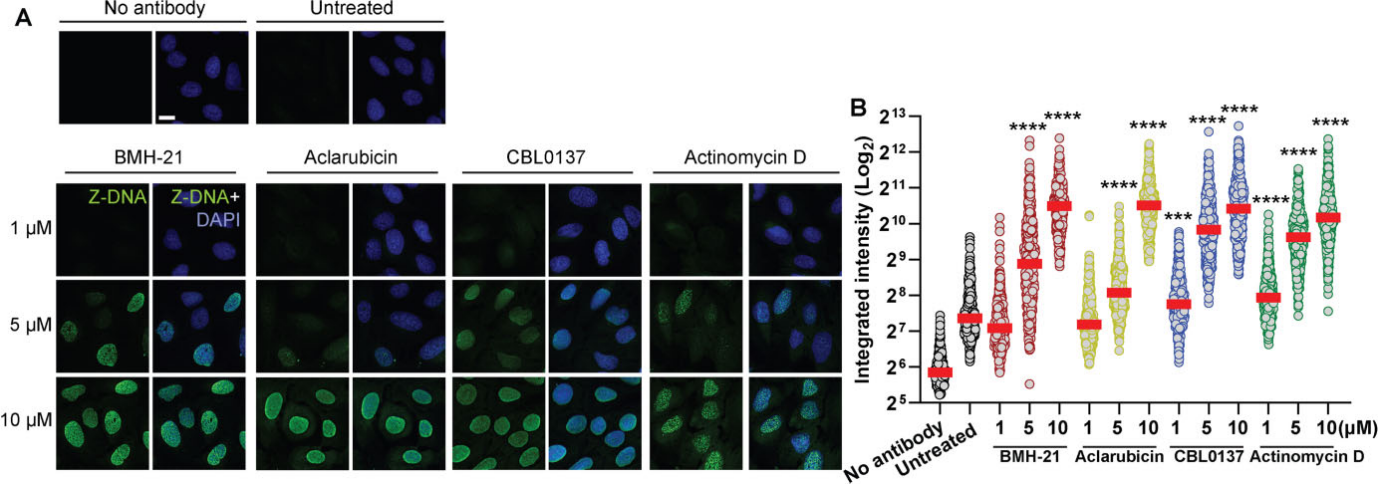
DNA intercalators induce the formation of Z-DNA. (**A**) Representative images and (**B**) signal quantification of immunofluorescence analysis of Z-DNA in U2OS cells treated with increasing concentrations of BMH-21, Aclarubicin, CBL0137 and Actinomycin D for 90 minutes. Scale bar 10 μm. Integrated intensity (arbitrary units) was analyzed (*n* > 300 cells per condition) by one-way ANOVA test compared to untreated, ***P value < 0.001, ****P value < 0.0001.

CBL0137 has been shown to induce a transition from B-DNA to Z-DNA (38). Z-DNA is the left-handed helical form of DNA in which the double helix winds to the left in a zigzag pattern. It differs from the canonical B-DNA that represents the main backbone of the human genome (47). We observed that BMH-21, Aclarubicin and Actinomycin D are also capable of inducing Z-DNA at high concentrations, as CBL0137 does (5–10 μM), with a progressive accumulation of pan-nuclear signal (Figure 7A and B). These results show that DNA intercalators progressively induce the accumulation of topological stress across the nuclear chromatin, likely explaining the collapse of the transcription machinery in the absence of DNA damage.

### Loss of POLR2A is more cytotoxic than loss of POLR1A and POLR3A

**Figure 8.**
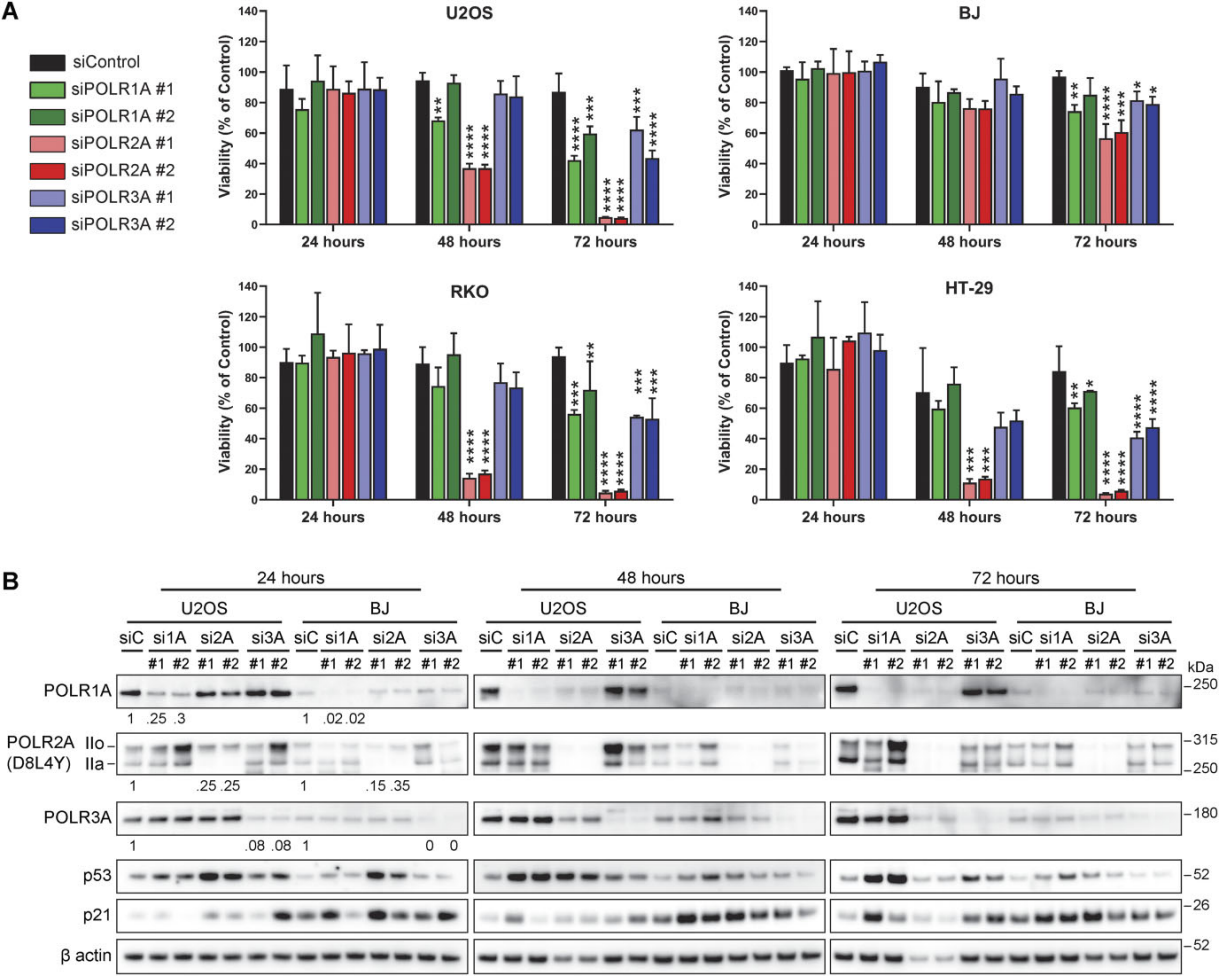
Knockdown of *POLR2A* impact cell viability more than the knockdown of *POLR1A* and *POLR3A*. (**A**) Cell viability analysis on U2OS, BJ, RKO and HT-29 cells after treatment with two oligos for siPOLR1A, siPOLR2A and siPOLR3A using Cell Titer Glo, after 24, 48 and 72 h of incubation. Data was analyzed by one-way ANOVA test compared to siControl for each timepoint (*n* = 3). *P value < 0.05, **P value < 0.01, ***P value < 0.001, ****P value < 0.0001. (**B**) Immunoblot analysis protein levels of RNA Pols, p53 and p21 in U2OS and BJ cells treated as in (**A**). The figure is representative of three similar and independent experiments. Signal levels of Pols I, II and II at 24 h after knockdown were quantified and normalized. Values under immunoblots at timepoint 24 h represent ratios of POLR1A, POLR2A (IIo + IIa) and POLR3A levels compared to siRNA Control (siC) and normalized using their respective β actin signal.

The three RNA polymerases are essential enzymes for cell survival and are often dysregulated in cancer, and therefore, they are interesting targets for therapy (48). Following our observations that DNA intercalators are inhibiting the activity and/or destabilizing the protein levels of all three polymerases, we wondered what is the contribution of each RNA polymerase to the maintenance of cell viability. To this end, we depleted each RNA polymerase using two siRNA oligos per gene for 24, 48 and 72 h in U2OS, BJ, RKO and HT-29 cell lines. Knockdown of POLR2A reduced viability to 40% in U2OS and to <20% in RKO and HT-29 at 48 h, with most cells dead by 72 h, while in siPOLR1A and siPOLR3A >40–50% of cells were viable at 72 h (Figure 8A). In BJ fibroblasts, siPOLR2A affected viability more than siPOLR1A and siPOLR3A, however, >50% of viability was still observed for all RNA polymerases at 72 h, in contrast to U2OS, RKO and HT-29 (Figure 8A). When comparing protein levels for each RNA polymerase in U2OS and BJ fibroblasts, we observed a reduction at 24 h (>70%) and most protein absent by 48 h (Figure 8B). Interestingly, siPOLR2A at 48 h negatively affected the protein levels of POLR1A and POLR3A, likely because Pol II transcribes the mRNAs for POLR1A and POLR3A. This is compatible with the reduction of β actin protein observed for siPOLR2A at 48 and 72 h in both cell lines, showing that targeting Pol II will have a rather fast effect on its target genes. Moreover, the knockdown of POLR2A had the strongest effect on p53 stabilization at 24 h in both cell lines (Figure 8B) and decreased its levels around 48 and 72 h. A possible explanation for the stabilization of p53 after Pol II inhibition is the downregulation of MDM2 expression, however, evidence shows that this p53 stabilization does not require the breakage of the p53-MDM2 regulatory loop (49). Knockdown of POLR1A starts to stabilize p53 at 24 h and this effect becomes stronger at 48–72 h, a process previously shown to be regulated through the impaired ribosome biogenesis checkpoint (35). Of interest is that POLR3A knockdown had a clear negative effect on viability at 72 h ranging from ∼40–80% in all tested cell lines, but without a major influence on p53 or p21 expression (Figure 8B). In this setting, recall that RNA Pol III plays a role in transcribing 5S rRNA, an integral part of the 5S RNP complex, and reduced 5S rRNA was shown to attenuate p53 stabilization (50).

Although a limitation of simulating the targeting of RNA Pols through siRNAs is not having a clear assessment of the interplay between basal protein levels, protein turnover and levels of transcription, as well as differences in rates of knockdown efficiency between cell lines, these results confirm and establish that targeting POLR2A levels has a faster and larger impact on cell viability than targeting POLR1A and POLR3A separately. In the context of DNA intercalators affecting the activity of the three enzymes, our results show that all three RNA Pols contribute to cell viability, therefore a compounding cytotoxic effect could be expected when using DNA intercalators. However, this finding does not take into consideration the cytotoxic potential of affecting DNA replication or other chromatin proteins such as FACT, TOPO II, histones, or other factors.

## Discussion

Targeting of Pol II and its transcription machinery has been proposed for the treatment of cancer (14,51,52). Here, we have shown that interfering specifically with Pol II has a greater cytotoxic effect than targeting Pol I or III separately. However, we have also shown that DNA-binding drugs impact the stability of all three nuclear RNA polymerases and generate additional effects on other chromatin factors and enzymes. Therefore, a compounding cytotoxic effect should be expected for DNA intercalators. In fact, we have known for some time that chemotherapeutical drugs that target DNA are also capable of interfering with rRNA transcription and processing, causing ribosome biogenesis stress that can contribute to their cytotoxic activity (53). This shows that DNA intercalators can affect many processes simultaneously, making it difficult to assess the separate contribution of each process to cell viability. This idea becomes more nuanced with the evidence that Pol II has a more direct role in ribosome biogenesis, contrary to previous notions indicating Pol I as the exclusive enzyme in charge of nucleolar transcription (54).

BMH-21, Aclarubicin and CBL0137 have quite different natural histories from discovery to validation. In this study we reveal that they affect chromatin biology through strikingly similar mechanisms. We propose, as others before (3,7), that more attention should be paid to DNA intercalators as drugs with a wide impact on chromatin dynamics, beyond seemingly specific effects on chromatin factors and enzymes. Therefore, the study of their cytotoxicity should take into consideration the effect of damaging essential processes taking place simultaneously throughout the chromatin network.

Here, we have shown that DNA-binding drugs induce degradation of Pol II in the absence of DNA damage. So far, most of the research around Pol II degradation has relied on the studies of DNA lesions induced by UV light and other sources of DNA damage (26,55). Pol II regulation is highly dynamic, with a series of sequential steps required to initiate, elongate and terminate mRNA synthesis (56). When Pol II encounters regions with damaged DNA, the enzyme is stalled, and a process of removal is set up in place. This leads to the ubiquitination of the enzyme, transportation by p97 and final degradation in the proteasome (57). Here we reveal that these DNA-binding drugs also interfere with chromatin positioning of Pol II, in the absence of DNA damage. This suggests the existence of another type of DNA lesion that may originate from torsional stress and that requires it to be resolved. The removal of RNA Pols from chromatin, along with the simultaneous recruitment of Topo II and FACT, suggests the need to repair topological issues, such as the progressive accumulation of Z-DNA.

The dysregulation of ribosome biogenesis has been increasingly recognized as a critical factor in cancer development and progression. Cancer cells, being exceptionally proliferative and metabolically demanding, place enormous stress on their ribosome production machinery. Consequently, targeting ribosome biogenesis in cancer presents a unique opportunity to disrupt the cellular processes vital for tumor growth and survival (13). Numerous well-known clinically used compounds and a few novel candidates have been thoroughly investigated as potential inhibitors of rDNA transcription, including Actinomycin D, BMH-21 and CX-5461 (11). In our hands, CBL0137 and Aclarubicin both exhibit effective inhibition of rDNA transcription, resulting in rapid nucleolar disruption, like BMH-21 and Actinomycin D do. This should be taken into consideration when interpreting results and clinical effects using CBL0137 and Aclarubicin. BMH-21 may specifically target and exploit unique vulnerabilities of Pol I during transcription elongation, without affecting the stability of the elongation complexes (58). Indeed, BMH-21 (15) and drugs such as Amodiaquine (19) and Aminacrine (59), can induce the degradation of POLR1A, the catalytic subunit of Pol I. Although all these aforementioned compounds readily inhibit Pol I transcription, the degradation of the enzyme has gained attention as a rather specific feature of a subset of these compounds (18). Despite the exceptional ability of BMH-21 to trigger POLR1A degradation, we observed that BMH-21 also has a significant impact on Pol II machinery. Upon treatment with a DNA intercalator, Pol II pool begins to be consumed, first with the disappearance of the unphosphorylated forms (IIa) and later with the decrease of the hyperphosphorylated form (IIo). This process requires the enzyme to be positioned on the chromatin in order to be labeled for degradation. Therefore, caution is necessary when interpreting biological phenotypes and solely attributing them to ribosome biogenesis inhibition. Moreover, the dynamic interplay between Pol I and Pol II in ribosome biogenesis further complicates the interpretation of results (54). However, our experiments with siRNA depletion of Pol I demonstrated that BMH-21′e effects on Pol II are not due to compensatory mechanisms.

Cumulative evidence has shown how Aclarubicin and CBL0137 impact chromatin fitness. Aclarubicin is an anthracycline structurally related to Doxorubicin but lacks its capacity to poison Topo II and therefore does not induce Topo II-dependent DNA damage. However, Aclarubicin retains its DNA intercalation activity, inducing histone eviction and resulting in epigenetic and transcriptomic alterations (60). We found that Aclarubicin also induces trapping of FACT and Topo II on chromatin. Another similarity described previously for CBL0137 and Aclarubicin is that both suppress the activity of the transcription NF-kB independently of DNA damage (61,62). Aclarubicin is classified as a catalytic inhibitor of Topo II (43) since it antagonizes the cytotoxicity of the Topo II poisons Etoposide (44) Doxorubicin (63) and Daunorubicin (64). Here, we show that BMH-21 also prevents Topo II activity by antagonizing the Topo II poisons Doxorubicin and Etoposide, therefore mimicking the effect of true catalytic inhibitors of Topo II. Although the use of Aclarubicin is discontinued world-wide for unclear reasons, it is used for the treatment of acute myeloid leukemia in Japan and China (7,65), indicating that a DNA intercalator can be used in therapy without the need to induce DNA damage.

Interestingly, we noted that the compound CX-5461 induced a reduction in POLR1A at high concentrations above 2 μM in U2OS cells (Supplementary Figure S1A, B). CX-5461 is known to effectively inhibit rDNA transcription, induce DNA damage, stabilize G-quadruplex structures, and enhancing radiosensitivity as a TOP2 inhibitor (29,66,67). More specifically, CX-5461′s effect on rDNA involves the inhibition of transcription by arresting Pol I within the transcription initiation complex, and as reported CX-5461 did not significantly affect transcription elongation *in vitro* suggesting that TOP2 poisoning may not be related to inhibition of rRNA synthesis (68).

CBL0137 was identified as a compound capable of trapping FACT on chromatin (61) and further characterized as and inducer of torsional stress by accumulation of Z-DNA (38). Furthermore, in a proteomic study that elucidated mechanisms of action, the activity of CBL0137 clustered together with transcription inhibitors such as Flavopiridol, a CDK9 inhibitor that inactivates Pol II (40), an effect that we have corroborated in our present study. In addition, CBL0137 is capable of triggering necroptosis through the activation of ZBP1 (Z-form nucleic acid binding protein 1) (69). We observed that BMH-21, Aclarubicin and Actinomycin D are also capable of inducing Z-DNA at high concentrations (5–10 μM) (Figure 7). These results show that DNA intercalators progressively induce the accumulation of topological stress across the nuclear chromatin, likely explaining the collapse of transcription machinery in the absence of DNA damage. However, the formation of Z-DNA can be associated with mutagenic processes under certain circumstances. When DNA undergoes structural changes, such as transitioning from B-DNA to Z-DNA, it can create strain and affect the stability of the DNA molecule. This structural instability could potentially increase the susceptibility of DNA to damage, such as breaks or base modifications, which may lead to mutations (70). This should be taken into consideration when evaluating the safety of ‘non-genotoxic’ DNA intercalating agents.

Assigning a mechanism of action to a DNA-binding drug is challenging. The concept of chromatin damage emerged to explain the cumulative amount of observations linking DNA intercalators and chromatin destabilization in the absence of DNA damage (3). Here, we do not propose a mechanism of action for DNA intercalators, but instead, we acknowledge the challenge of disambiguating the cellular mechanism of toxicity when the whole chromatin network is being destabilized simultaneously. An example of this challenge is the drug CX-5461, a compound originally developed from precursors capable of interfering with Top II and interacting with G-quadruplex (71), which was further shown to target Pol I activity (72) but also having a larger cytotoxicity on cells with BRAC1/2 deficiency (29) and it is a powerful mutagen (73). It was shown that its capacity to induce DNA damage was at least in part through Topo II poisoning (28). An intriguing aspect in the development of CX-5461, BMH-21, Aclarubicin and CBL0137 is that, at some point, other compounds in their series also moved between having or lacking the capacity to induce DNA damage. During the development of CX-5461, its precursor QQ58 behaved as a catalytic inhibitor of Topo 2, while A-62176, the precursor of QQ58, was characterized as a Topo II poison (71). In the development of BMH-21, its related compounds BMH-7 and BMH-15 enabled an ATM-dependent phosphorylation of H2AX, a marker of DNA damage (74). As a member of the anthracyclines, Aclarubicin probably exemplifies the first case where a change in structure rendered the compound incapable of poisoning Topo II as its analog Doxorubicin (7). As well, during the development of CBL0137 and although not structurally related to curaxins, authors also noted that 9-aminoacridine (9AA) and Quinacrine do not induce DNA damage, while the 9AA derivate Amsacrine is known for being a Topo II poison (43,75).

Evidence shows that cell death induced by Topo II poisons requires the presence and activity of Topo II, since the proteins involved in resolving Topo II cleavage complexes rank among the genes that confer the highest resistance to Topo II poisoning (e.g. ZNF451, TDP2, LIG4) (76). However, in the absence of poisoning activity, DNA intercalators still induce cell death and impact genome biology through mechanisms distinct from DNA damage (3,7,9). Given the great number of chemical structures capable of DNA intercalation and Topo poisoning (30,77), and the aforementioned structural relationship between both activities within drug series (34), these types of compounds are likely to still emerge from chemical screens or similar studies, therefore, attention should be paid to study and validate their features in a comprehensive way.

## Supplementary Material

gkae069_Supplemental_File

## Data Availability

The data underlying this article are available in the article and in its online supplementary material. All original raw immunofluorescence images and full scans of immunoblots used in this article are available upon request.
