# Supplementary material for: Chromatin damage generated by DNA intercalators leads to degradation of RNA Polymerase II
Source: Nucleic Acids Res. 2024 Feb 10;52(8):4151–66. doi: 10.1093/nar/gkae069 (PMC11077059; doi:10.1093/nar/gkae069)

Supplementary information

Chromatin damage generated by DNA intercalators leads to degradation of RNA Polymerase II

Jaime A. Espinoza[1#], Dimitris C. Kanellis[1], Sheetanshu Saproo[1], Karla Leal[1], Johana Fernandez Martinez[1], Jiri Bartek [1,2#] & Mikael S. Lindström[1#]

[1] Science for Life Laboratory, Division of Genome Biology, Department of Medical Biochemistry and Biophysics, Karolinska Institutet, S-171 21 Stockholm, Sweden

[2] Danish Cancer Society Research Center, DK-2100 Copenhagen, Denmark

# Correspondence

Supplementary Figure 1. (A) Immunoblot analysis of protein levels of POLR1A, POLR2A, POLR3A, MYC, p53 and yH2AX in U2OS cells treated with chemotherapeutic drugs for 4 hours (**B**) Immunoblot analysis of POLR1A in U2OS and BJ fibroblasts treated with increasing concentrations of CX-5461 for 4 hours. (**C**) RT-qPCR analysis of *POLR1A*, *POLR2A*, *POLR3A*, *MYC* mRNAs and *47S* rRNA in BJ fibroblasts, HT29 and RKO cells. Data are presented as the mean ± SD (n=3), *= P value < 0.05, **= P value < 0.01, ***= P value < 0.001 by Student's t test versus untreated cells (**D**). RT-qPCR analysis of the mRNA levels for the twelve subunits of II and *47S* rRNA in U2OS cells treated with BMH-21 (1 µM), Aclarubicin (1 µM) and CBL0137 (1 µM) for 3 hours. Data are presented as the mean ± SD (n=3), *= P value < 0.05, **= P value < 0.01, ***= P value < 0.001 by Student's t test versus untreated cells (**C**).

Supplementary Figure 2. (**A**) Immunofluorescence images of representative cells stained with Fibrillarin and Nucleophosmin primary antibodies after treatment with BMH-21, Aclarubicin, CBL0137 and Actinomycin D for 4 hours. Scale bar 5 µm. (**B-C**) Immunofluorescence images of representative cells stained with 5.8S rRNA and POLR1A primary antibodies after treatment with BMH-21 and Aclarubicin in U2OS and BJ fibroblasts cells. Scale bar 5 µm. White arrows show nucleolar regions.

Supplementary Figure 3. (**A**) Immunoblot analysis of POLR1A, POLR2A (D8L4Y) and POLR3A in HT29, RKO, BJ, A375 and LoVo cells treated with 1 µM BMH-21 for 3 hours. (**B-E**) Immunoblot analysis of BJ Fibroblasts whole cell lysates and subcellular fractions treated with increasing concentrations of (**B**) BMH-21, (**C**)

Aclarubicin, (**D**) CBL0137 and (**E**) U2OS cells treated with Actinomycin D. Values under immunoblots represent ratios of POLR1A levels compared to concentration 0 and normalized using their respective Lamin A/C signal. (**F**) Immunoblot analysis of POLR3A and UBF in in whole cell lysate and cellular fractionations of U2OS cells treated with BMH-21 (0.5 and 5 μM), Aclarubicin (5 μM) and CBL0137 (5 μM) for 3 hours.

Supplementary Figure 4. (**A**) Immunoblot analysis of phophoSer139-H2AX in U2OS cells pre-treated with growing concentrations (0.1-1 μM) of BMH-21 for 1 hour and further treated with 1 μM Doxorubicin and 1 μM Etoposide for 3 hours. (**B**) Immunoblot analysis of phophoSer139-H2AX in U2OS pre-treated with Actinomycin D and Triptolide and further treated with 1 μM of Doxorubicin and Etoposide for 3 hours.

Supplementary table S1. Cell lines

Cell line	Medium
U2OS	DMEM 10% FBS + Pen/strep
BJ fibroblasts	DMEM 10% FBS + Pen/strep
RKO	DMEM 10% FBS + Pen/strep
Lovo	DMEM 10% FBS + Pen/strep
HT29	DMEM 10% FBS + Pen/strep
A375	DMEM 10% FBS + Pen/strep

Supplementary table S2. Antibodies

Antibodies	Company	Clone	Catalogue No	Application (dilution)
POLR1A	Cell Signalling Technology	D6S6S	24799	WB (1:2,000)<br>IF (1:500)
Rpb1 NTD (POLR2A)	Cell Signalling Technology	D8L4Y	14958	WB (1:2,000)<br>IF (1:500)
POLR2A-phospho-CTD-Ser-5	EMD Millipore	3E8	04-1572	WB (1:4,000)
POLR2A-phospho-CTD-Ser-2	EMD Millipore	3E10	04-1571	WB (1:4,000)<br>IF (1:500)
POLR2A	Novus Biologicals	4H8	NB200-598	WB (1:4,000)
POLR3A	Cell Signalling Technology	D5Y2D	12825	WB (1:2,000)
TOP2A (Topo II Alpha)	Bethyl Laboratories	Polyclonal	A300-054A	WB (1:2,000)
TOP2B (Topo II Beta)	Bethyl Laboratories	Polyclonal	A300-950A	WB (1:2,000)
TOP1 (Anti-Topoisomerase I)	Abcam	EPR5375	ab109374	WB (1:2,000)
Double-stranded DNA	Abcam	35I9	Ab27156	Slot blot (1:4000)
SPT16	Cell Signalling Technology	D7I2K	12191S	WB (1:2,000)
SSRP1	Cell Signalling Technology	E1Y8D	13421S	WB (1:2,000)
Lamin A/C	Santa Cruz Biotechnology	H-110	sc-20681	WB (1:2,000)
Histone H2A	Abcam	EPR17470	ab177308	WB (1:2,000)
Histone H2B	Abcam	H2BC12	ab52484	WB (1:2,000)
Histone H2A.X	Merck Millipore	Polyclonal	07-627	WB (1:2,000)
Phospho-H2A.X (ser139)	Millipore	JBW301	05-636	WB (1:4,000)<br>IF (1:500)
Histone H3	Merck Millipore	Polyclonal	06-755	WB (1:2,000)
Histone H4	Merck Millipore	Polyclonal		WB (1:2,000)
Vinculin	Abcam	EPR8185	ab129002	WB (1:2,000)
p53	Abcam	DO-1	ab1101	WB (1:2,000)
p21	Cell Signalling Technology	12D1	2947	WB (1:2,000)
z-DNA	Absolute Antibody	Z22	Ab00783-3.0	IF (1:200)
5.8S rRNA	Novus Biologicals	Y10b	NB100-662	IF (1:500)
c-Myc	Cell Signalling Technology	D84C12	5605	WB (1:2,000)
β-actin	Abcam	AC-15	ab6276	WB (1:20,000)
Fibrillarin	Abcam	Polyclonal	ab5821	IF (1:500)
Nucleophosmin	Abcam	FC82291	Ab10530	IF (1:500)
Anti-mouse Alexa-647	Thermo	Polyclonal	A-21235	IF (1:5000)
Anti-mouse Alexa-488	Thermo	Polyclonal	A-11029	IF (1:5000)
Anti-rabbit Alexa-647	Thermo	Polyclonal	A-21244	IF (1:5000)
Anti-rabbit Alexa-488	Thermo	Polyclonal	A-11008	IF (1:5000)

Supplementary table S3. Chemicals

Chemical	Company	Catalogue code	Solvent
BMH-21	Sigma-Aldrich	SML1183	DMSO
CBL0137	Cayman Chemical Company	19110	DMSO
Aclarubicin	Cayman Chemical Company	15993	DMSO
Actinomycin D	Sigma-Aldrich	A1410	DMSO
Triptolide	Tocris	3253	DMSO
Doxorubicin	Sigma-Aldrich	D1515	DMSO
Etoposide	TopoGen	TG4140	DMSO
Camptothecin	Sigma-Aldrich	C9911	DMSO
Topotecan	Sigma-Aldrich	T2705	DMSO
Mitoxantrone	Sigma-Aldrich	M6545	DMSO
Neocarzinostatin	Sigma-Aldrich	N9162	DMSO
CB-5083	Selleck Chemicals	S8101	DMSO
MLN-4924	Selleck Chemicals	S7109	DMSO
THZ1	Selleck Chemicals	S7549	DMSO
Flavopiridol	Cayman Chemical Company	10009197	DMSO
MG-132	Selleck Chemicals	S2619	DMSO
CX-5461	Selleck Chemicals	S2684	50 mM NaH2PO4
Oxaliplatin	Sigma-Aldrich	Y0000271	Water
4-Nitroquinoline N-oxide (4-NQO)	Sigma-Aldrich	N8141	DMSO
5-Fluorouracil	Sigma-Aldrich	F6627	DMSO
Metarrestin	MedChem Express	HY-120118	DMSO

Supplementary table S4. RT-qPCR primer sequences

Target	Forward	Reverse
47S_5´ETS*	GAACGGTGGTGTGTCGTT	GCGTCTCGTCTCGTCTCACT
18S_5'-junction*	GCCGCGCTCTACCTTACCTACCT	CAGACATGCATGGCTTAATCTTTG
18S_3'-junction*	AGTCGTAACAAGGTTTCCGTAGGT	CCTCCGGGCTCCGTTAAT
POLR1A	TTTGCCGTGTATGGCATCGC	TGTCATCTGCTGTAGCGGGG
POLR2A	TCAAGAACTAGTGCGCAGGG	TGGAAACGCAAGTCAATGCG
POLR3A	GACTTAAAGCCCAGCCAGGT	GGCTCATCGTCAGGTGTGAA
MYC	CCCTCCACTCGGAAGGACTA	GCTGGTGCATTTTCGGTTGT
ACTB	TCACAATGTGGCCGAGGACTTT	AGAAGTGGGGTGGCTTTTAGGA
QARS	ACCTGAACCTGGCATCACTACA	CCAAGACGCTCAAACTGGAACT
POLR2B	GCTTCTGGGCGGTTTTTGTC	CCGCGTCGTACATATTGCCA
POLR2C	CGGAGCTCACTGACGAGAAT	CTCAGCGATGAAGACCCTCC
POLR2D	TCTAAGCCTCTGGCCTGCTA	GCAGTGGCTCAAGAGTGGAT
POLR2E	CTGGCCCGATATAAGCTCCG	TGCCCACGCTTTATCCCAAA
POLR2F	ATGACCAAGTACGAGCGAGC	AGCAGAGGATCTGTCTCCCC
POLR2G	GGACCCGTGTGGACAAGAAT	TAGGACCAAGGGTAGGAGGC
POLR2H	TTGTTCAAGCCTGAGTGGCA	TCACAGGCGAGTCAGTTTCC
POLR2I	AGATTATCGCCGACGTGTCC	AGAACACAGCCTCCTTGTGG
POLR2L	GACTGGGCCATGAACTCTCC	GGAGGGAACCTCAAAGAGCC
POLR2J	CACCCCTTGGAGCACAAGAT	TCGGTGATGGCGTTGGTAAA
POLR2K	TTGGAAACGCGGAGTGAGTT	GCTGCTTTGGAGGTTGAACG

*Sequences obtained from Kwon *et al*, 2014 (22).

Supplementary Figure 1

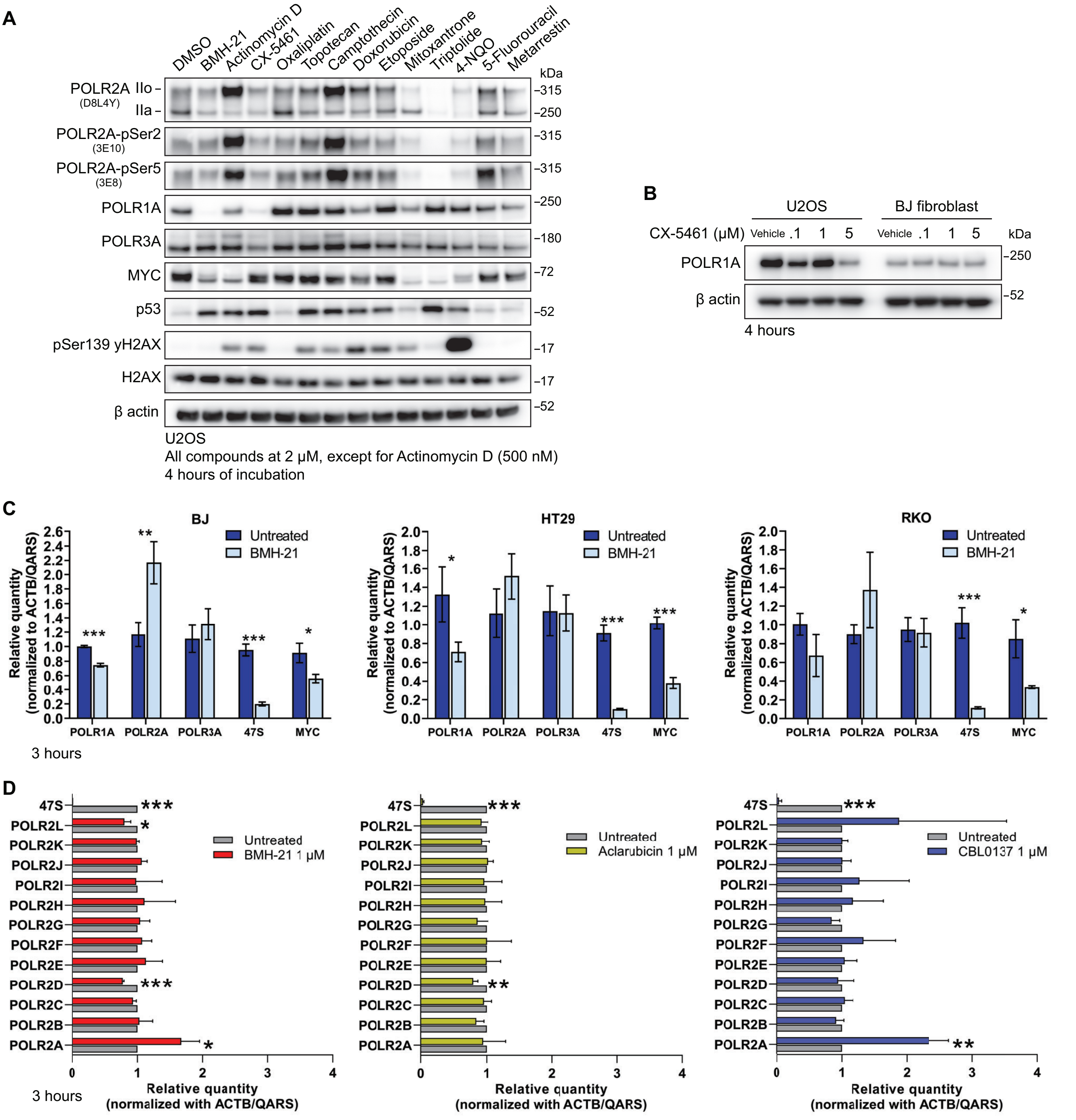

Supplementary Figure 2

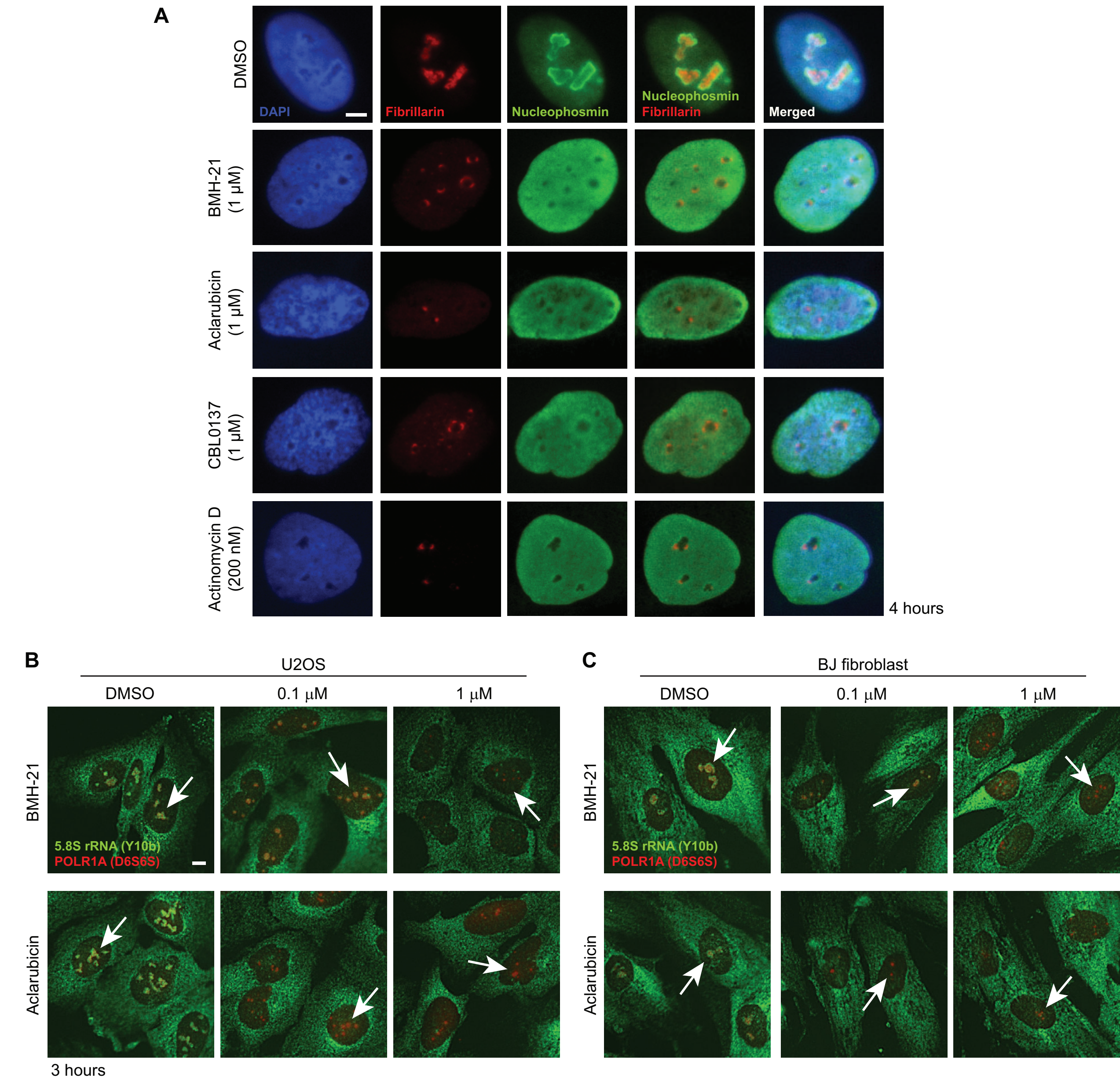

Supplementary Figure 3

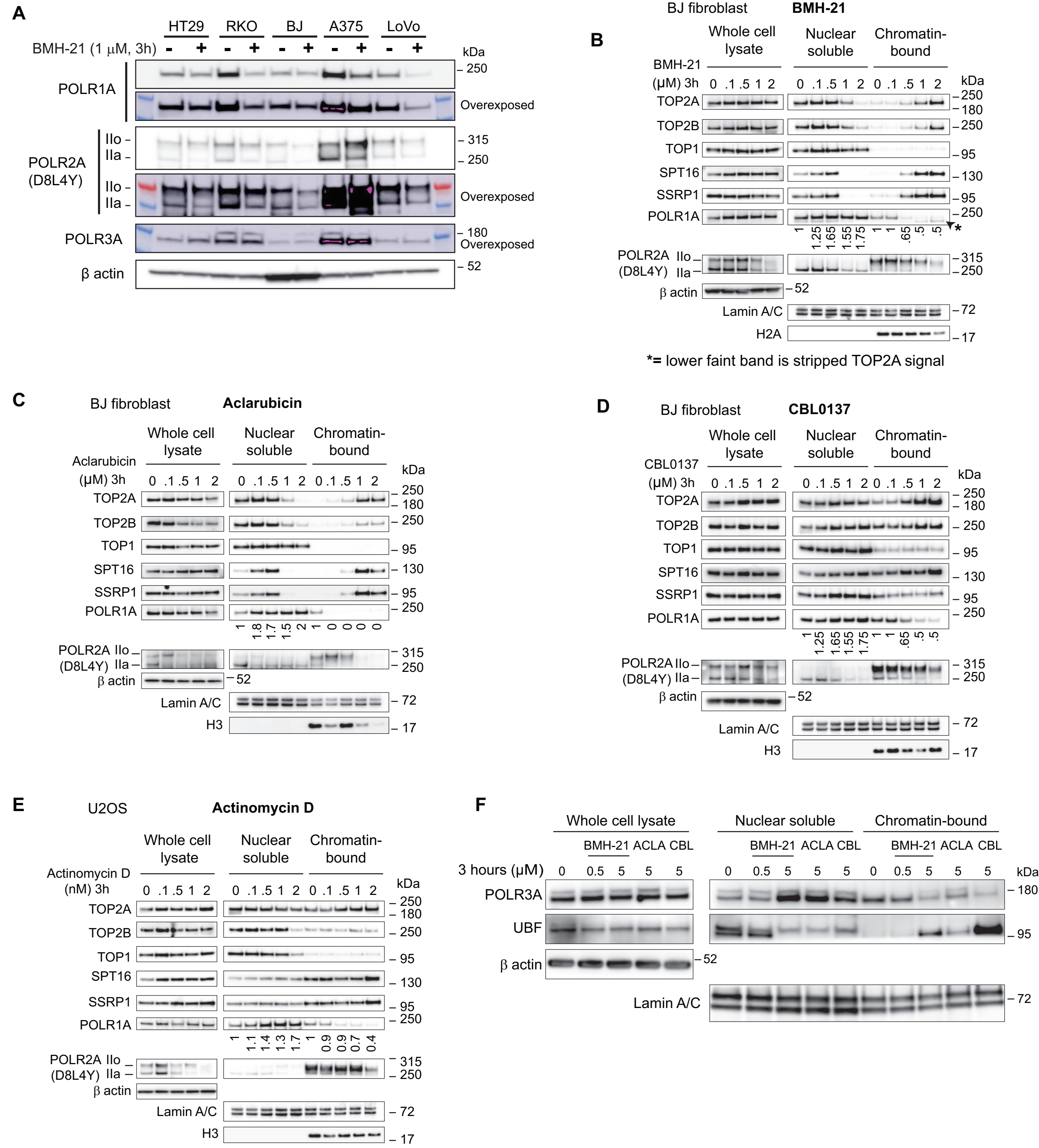

Supplementary Figure 4

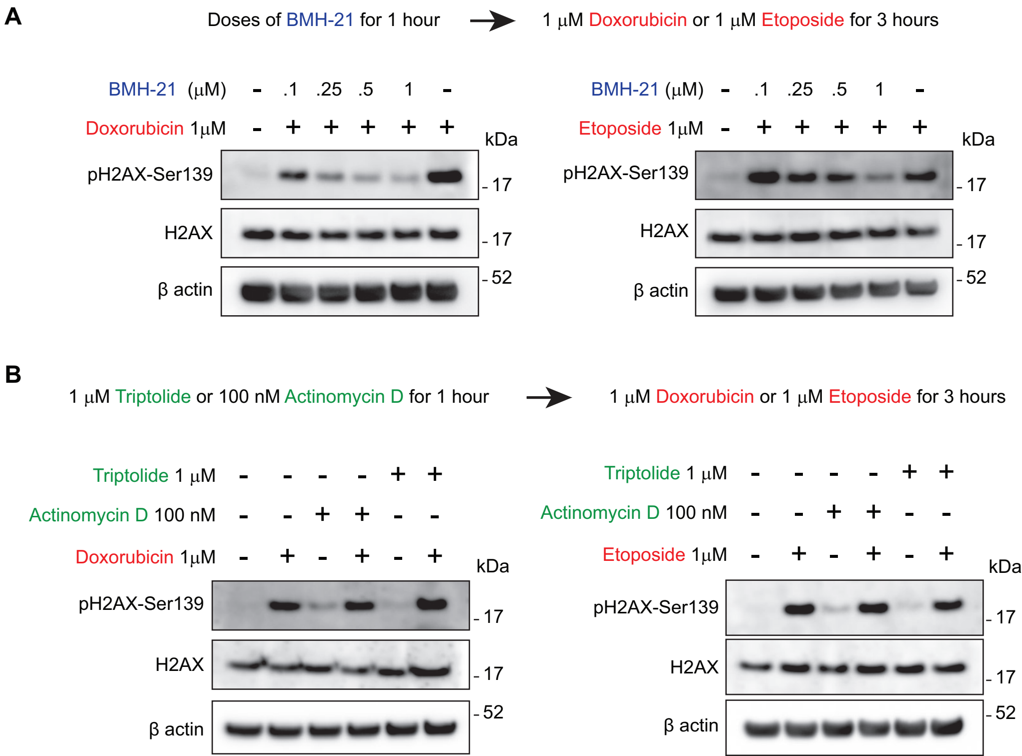

Supplement: gkae069_Supplemental_File [file gkae069_supplemental_file.pdf]
